# Role of Peripheral Immune Cells for Development and Recovery of Chronic Pain

**DOI:** 10.3389/fimmu.2021.641588

**Published:** 2021-02-22

**Authors:** John R. Bethea, Roman Fischer

**Affiliations:** ^1^Department of Biology, Drexel University, Philadelphia, PA, United States; ^2^Institute of Cell Biology and Immunology, University Stuttgart, Stuttgart, Germany; ^3^Stuttgart Research Center Systems Biology, University of Stuttgart, Stuttgart, Germany

**Keywords:** chronic neuropathic pain, immune cells, T cells, Tregs, recovery, macrophages

## Abstract

Chronic neuropathic pain (CNP) is caused by a lesion or disease of the somatosensory nervous system. It affects ~8% of the general population and negatively impacts a person's level of functioning and quality of life. Its resistance to available pain therapies makes CNP a major unmet medical need. Immune cells have been shown to play a role for development, maintenance and recovery of CNP and therefore are attractive targets for novel pain therapies. In particular, in neuropathic mice and humans, microglia are activated in the dorsal horn and peripheral immune cells infiltrate the nervous system to promote chronic neuroinflammation and contribute to the initiation and progression of CNP. Importantly, immunity not only controls pain development and maintenance, but is also essential for pain resolution. In particular, regulatory T cells, a subpopulation of T lymphocytes with immune regulatory function, and macrophages were shown to be important contributors to pain recovery. In this review we summarize the interactions of the peripheral immune system with the nervous system and outline their contribution to the development and recovery of pain.

## Introduction

Chronic pain is defined as a more than 12 weeks lasting pain that is characterized by irregular somatosensory processing in the peripheral nervous system (PNS) or the central nervous system (CNS) (1). Chronic neuropathic pain (CNP) is a specific form of chronic pain that is caused by damage to or disease of the somatosensory nervous system (2, 3) and affects up to 8% of the general population (4). CNP can result as a consequence of a large number of medical conditions, such as injuries to the PNS or CNS, metabolic, autoimmune or neurodegenerative diseases as well as cancer and chemotherapy (4, 5). In this review, we will mainly focus on CNP due to peripheral nerve injuries and discuss results from chemotherapy-induced pain. Individuals suffering from CNP exhibit stimulus-independent pain that is often characterized by abnormal sensations or hypersensitivity in the affected area. Patients often describe the pain as a burning and/or stabbing sensation (6). Allodynia, pain elicited by a usually non-painful stimulus, and hyperalgesia, an increased pain response due to painful stimulus, are frequent symptoms described by CNP patients (7). CNP can have a dramatic impact on a person's level of functioning and quality of life and is resistant to conservative pain management (7). Therefore, effective therapies that either prevent or reverse CNP are critical for both public health and clinical practice (5).

Research from the last two decades has demonstrated that a robust neuroimmune response and bidirectional signaling between the sensory and immune system contribute to development and maintenance of CNP. Indeed, increased levels of soluble pro-inflammatory mediators and recruitment of immune cells to the site of nerve injury, the dorsal root ganglion (DRG) and the spinal cord after PNS/CNS injury are well-characterized in rodent models of CNP (8–15). In addition, immune cells also contribute to recovery of CNP (16–18), indicating that modulation of immunity has therapeutic potential to treat CNP. Therefore, a detailed understanding of the contribution of immune responses to the development, maintenance and resolution of CNP may result in novel therapeutic approaches that are superior to current pain-relieving therapies.

## Development and Maintenance of CNP is Controlled by CD4^+^ Effector T Cells

T cells were identified as important contributors to CNP development and maintenance in animal models and human patients. Early on, the general importance of T cells for pain development was demonstrated by several independent studies showing that immunodeficient mice without functional lymphocytes in contrast to control mice do not develop pain hypersensitivity after nerve injury (9, 19, 20). Reconstitution of immunodeficient nude rats (9), and mice (19) with CD4^+^ T helper cells resulted in reestablishment of the pain phenotype, suggesting that pro-inflammatory T helper cells promote pain responses. T cells were shown to infiltrate into the lumbar spinal cord post-injury (19). Similar, the Th1 cytokine interferon-γ (IFN-γ) is upregulated in the dorsal horn after nerve injury and functional IFN-γ signaling is required for full development of neuropathic hypersensitivity (20), indicating that Th1 responses in the spinal cord contribute to pain hypersensitivity. The important role of T cell infiltration for development of pain was further confirmed by a recent study demonstrating that intrathecal injection of a T cell receptor (TCR) specific antibody that depletes functional CD4^+^ T cells resulted in alleviation of mechanical allodynia, indicating that CNS infiltrating T cells directly contribute to pain responses. Once treatment was terminated, mechanical allodynia returned to levels comparable to control mice, presumably due to repopulation of functional T cells in the CNS (21).

Next to IFN-γ expressing Th1 cells, IL17-expressing T helper cells were detected in peripheral nerves, indicating that Th17 cells may also contribute to pain development (22). Indeed, intrathecal and intraneural injection of recombinant IL-17A induced pain hypersensitivity (23), indicating a role of IL-17A for pain development. A recent study further confirmed that IL-17A regulates neuron-glial communications, synaptic transmission, and neuropathic pain after chemotherapy (24). Additional mechanistic data showed that DRG-infiltrating T lymphocytes release leukocyte elastase after nerve injury. Confirming that leukocyte elastase promotes T cell-dependent pain responses, adoptive transfer of leukocyte elastase deficient T cells did not restore pain development in immunodeficient Rag2^−/−^ mice (25). This indicated that T cells may also directly contribute to nerve damage.

Interestingly, MHC class II knockout (k/o) mice that lack MHC class II-restricted T helper cells displayed an impaired chronification of mechanical allodynia after peripheral nerve injury (26), indicating the general importance of CD4^+^ T helper cells for pain chronification in rodents. This was confirmed by a recent study showing that MHC class II-restricted CD4^+^ T helper cells contribute to the transition from acute to chronic mechanical allodynia in a rat model of peripheral nerve injury (21). Clinical studies have shown that a human major histocompatibility complex (MHC) class II gene polymorphism (DQB1^*^03:02 HLA haplotype) is associated with an increased risk to develop CNP after inguinal hernia surgery and lumbar disc herniation (27). The importance of MHC class II genes for CNP development indicates that, similar to the animal models, CD4^+^ Th cells contribute to CNP development in human patients. The relevance of MHCII-restricted T helper cells as an important trigger for chronic hypersensitivity after nerve injuries has been recently discussed by Ding et al. (28), where they indicate that there is a growing body of clinical evidences showing that increased blood Th cell numbers and changes in subset patterns are correlated with neuropathic pain intensities after nerve injuries. Other clinical data indicate that an emergent T-helper 2 profile with high interleukin-6 levels correlates with the appearance of bortezomib-induced neuropathic pain (29). Interestingly, Luchting et al. (30) found a disrupted Th17/Treg balance with significantly increased anti-inflammatory Tregs and decreased pro-inflammatory Th17 cells in patients suffering from chronic unspecific low back pain compared to healthy controls, indicating an anti-inflammatory T cell shift in the patients they analyzed. Altogether, these clinical data indicate that there is a considerable impact of the T cell compartment in neuropathic pain. However, the role of pro- and anti-inflammatory T cell subsets in CNP patients seems to differ depending on the pain condition of the patients.

## T Cell Subsets Contribute To Pain Recovery

In 2004 Moalem et al. (9) showed that adoptive transfer of Th2 polarized T helper cells in athymic nude rats further reduced pain hypersensitivity, indicating that anti-inflammatory responses mediated by Th2 cells may counteract inflammatory processes that promote pain development and maintenance. Indeed, higher circulating levels of the anti-inflammatory interleukins IL-10 and IL-4 were detected in patients with painless neuropathy compared to patients with painful neuropathy and controls (31), indicating that anti-inflammatory responses may be necessary to control pain development. Indeed, Leger et al. (32) demonstrated that glatiramer acetate treatment, an approved MS therapy, inhibited microglia activation and increased IL-10 and IL-4 expressing T cells in the dorsal horn after peripheral nerve injury resulting in alleviation of neuropathic allodynia. This indicates that next to their pathologic role for development and maintenance of CNP, T cells may also contribute to the resolution of pain.

## CD8^+^ T Cells Contribute To Pain Recovery

Whereas, CD4^+^ Th1 cells promote pain development and maintenance, several reports indicate that CD8^+^ T cells may contribute to pain resolution. Krukowski et al. (33) showed that chemotherapy-induced mechanical hypersensitivity was prolonged in T-cell-deficient Rag1^−/−^ mice compared to wild type mice. Adoptive transfer of CD8^+^, but not CD4^+^ T cells to neuropathic Rag1^−/−^ mice restored pain resolution. Mechanistically, this study indicated that CD8^+^ T Cells and endogenous IL-10 were required for resolution of CNP (33). Other studies have also reported a role of IL-10 for pain resolution and functional recovery after peripheral nerve injury (34), indicating the general importance of anti-inflammatory responses for pain resolution. Laumet et al. (35) recently confirmed that resolution of chemotherapy-induced mechanical allodynia is dependent on presence of CD8^+^ T cells. They showed that adoptive transfer of CD8^+^ T cells from naïve wildtype mice to T-cell-deficient neuropathic Rag2^−/−^ mice failed to promote pain resolution. In contrast, adoptive transfer of cisplatin-educated CD8^+^ T cells prevented the development of chemotherapy-induced CNP (35). Importantly, this T cell education appeared to be independent of antigen recognition by the T cell receptor because cisplatin-educated CD8^+^ T cells did also promote pain resolution in a model of paclitaxel-induced CNP and reconstitution of T cell deficient mice with ovalbumin-specific CD8^+^ T cells also restored CNP resolution (35). This study indicates that CD8^+^ T cells need to be activated to acquire the capacity to promote resolution of CNP, but their therapeutic activity seems to be independent of their antigen-specific education. However, the role of CD8^+^ T cells for CNP seems to be complex. Using a model of paclitaxel-induced CNP, Liu et al. (36) showed that blocking of functional CD8^+^ T cells at the level of the spinal cord and the DRG, reversed chemotherapy induced mechanical hypersensitivity. Similar, adoptive transfer of CD8^+^ T cells exacerbated neuropathic pain in this model (36), suggesting that cytotoxic T cells contribute to pain progression.

## Tregs Control Immunity to Promote Pain Resolution

Another important T cell subset that plays a role for pain recovery are regulatory T cells (Tregs), immunomodulatory T lymphocytes that control the activity of innate and adaptive immune cells (37). After peripheral nerve injury, Tregs are recruited to the site of injury, the DRG and the spinal cord (16, 17, 38). Systemic expansion of Tregs was shown to alleviate peripheral CNP following nerve injury and experimental autoimmune neuritis-associated central CNP (16). Similar, anti-CD25 antibody-dependent depletion of CD25^+^ cells prolonged mechanical hypersensitivity after peripheral nerve injury (16, 18), indicating a role of CD25^+^ Tregs for pain recovery. In another study DEREG mice, where FoxP3-expressing Tregs can be depleted by injection of diphtheria toxin, were used to specifically assess Treg contribution to pain recovery. Indeed, following toxin application DEREG mice developed increased mechanical pain hypersensitivity after peripheral nerve injury (17), confirming that Tregs are important for pain recovery. The analgesic role of Tregs was further confirmed in a model of chemotherapy-induced CNP, where adoptive transfer of a population of CD4^+^CD25^+^ T cells, which largely are composed of Tregs, alleviated CNP (36).

In a recent study it was shown that nerve-infiltrating Tregs suppress the development of neuropathic pain mainly through the inhibition of the Th1 response by CD4^+^ T helper cells following nerve injury (38). This resulted indirectly in reduced neuronal damage and neuroinflammation at the level of the sensory ganglia. The authors further identified IL-10 signaling as an intrinsic mechanism by which Treg cells counteract neuropathic pain development (38). Indeed, in a previous study the neuroprotective effect of IL-10 secretion by CNS infiltrating Tregs was demonstrated in an ischemia model (39). Another recent study by Duffy et al. (40) showed that adoptive transfer of activated Tregs or intrathecal delivery of the Treg cytokine IL-35 alleviated spontaneous and facial stimulus-evoked pain behaviors in mice with experimental autoimmune encephalomyelitis (EAE). The effects of intrathecal IL-35 therapy were dependent on presence of Tregs and associated with reduced monocyte infiltration in the trigeminal afferent pathway and upregulated IL-10 expression in CNS-infiltrating lymphocytes (40). Interestingly, intrathecal injection of plasmids encoding IL-10 at the onset of clinical EAE suppressed disease development and alleviated pain behaviors (41, 42), indicating that the upregulated IL-10 expression observed in the study of Duffy et al. (40) may be responsible for the observed pain alleviating effect of Tregs.

All these data point toward a critical role of IL-10 for pain resolution. Indeed, additional research showed that central activation of anti-inflammatory cytokines such as IL-10 and TGFβ suppresses allodynia after peripheral nerve injury (43) and in a model of chemotherapy-induced neuropathic pain (44). Mechanistically, *in vitro* studies showed that in addition to its master anti-inflammatory role, IL-10 reverses voltage-gated sodium currents to reduce neuronal excitability (45), indicating a possible immune cell independent mechanism of IL-10 mediated pain recovery. Therefore, therapies that promote Treg activity and IL-10 signaling in the CNS may prove beneficial for pain therapies.

Over the last decade, we and others showed that tumor necrosis factor receptor 2 (TNFR2) is critical for Treg function and that selective agonism of TNFR2 results in Treg expansion and is therapeutic in inflammatory conditions such as experimental arthritis (46, 47) and graft vs. host disease (48). We recently demonstrated that treatment of neuropathic mice with a TNFR2 agonist promoted long-term pain recovery after peripheral nerve injury (18) and in neuropathic EAE mice (49). Mechanistically, our study revealed that systemic TNFR2 agonist application promoted expansion of Tregs resulting in alleviation of peripheral and central inflammation. We further detected increased Treg and IL-10 levels in the spinal cord after TNFR2 agonist treatment (18), indicating that Treg-mediated IL-10 signaling in the CNS may contribute to the pain alleviating effect of TNFR2 agonists too. Interestingly, next to their anti-inflammatory functions, Tregs were shown to directly promote tissue regeneration in the CNS (50). Confirming, we observed upregulation of various proteins associated with neuroregeneration after TNFR2 agonist treatment in neuropathic mice (18), indicating that TNFR2-dependent expansion of Tregs may promote pain recovery not only by modulation of immunity, but also via enhanced tissue regeneration. Indeed, previous studies showed that TNFR2 directly contributes to neuroprotection (51–53).

## Macrophages Contribute to The Development of CNP

Next to T lymphocytes, peripheral monocytes that differentiate into macrophages upon tissue infiltration were shown to play a role for pain development. These cells were shown to infiltrate around injured sensory neurons and in the DRG. Inhibition of monocyte infiltration into the DRG prevented the development of pain hypersensitivity in rodent models of CNP (54, 55), indicating the importance of peripheral macrophages for pain development. In a model of chemotherapy-induced CNP, nerve infiltrating monocytes were activated by the chemokine fractalkine (CX3CL1) resulting in the production of reactive oxygen species that in turn activated the receptor TRPA1 in sensory neurons and evoked the pain response (56). Additional data indicate an interaction between the fractalkine receptor CX3CR1 and the chemokine receptor CCR2 in monocytes that may constitute an underlying mechanism for persistent chemotherapy-induced pain (57).

Monocytes/macrophages were shown to act synergistically with microglia to initiate hypersensitivity and promote the transition from acute to chronic pain after peripheral nerve injury (58). A recent study demonstrated that DRG macrophages, but not macrophages that had infiltrated at the site of injury contribute to initiation and maintenance of mechanical hypersensitivity (59). Indeed, depletion of DRG macrophages, but not at the site of injury, prevented the development of pain and reversed ongoing nerve injury-induced hypersensitivity (59). Macrophages that invade the DRG, release excitatory agents that generate ectopic activity in sensory neurons thereby contributing to neuropathology responsible for pain development (60, 61).

## Macrophages Alleviate Pain via The Opioid System and Anti-Inflammatory Responses

Macrophage infiltration into the nerve is an essential step to allow nerve regeneration. In particular, anti-inflammatory/reparative M2 macrophages have been indicated to play a role for repair processes after nerve injury (62). Indeed, perineural transplantation of M2 macrophages resulted in attenuated neuropathy-induced mechanical hypersensitivity (63, 64). Similar, injection of IL-4, a cytokine responsible for M2 macrophage differentiation, at the site of nerve injury promoted repolarization of macrophages into an anti-inflammatory M2 state, and ameliorated mechanic and thermal hypersensitivity (65). Recently, it was shown that local sympathectomy relieves chemotherapy-induced allodynia in mice via anti-inflammatory responses. Depletion of monocytes/macrophages and blockade of transforming growth factor-β (TGF-β) signaling reversed the relief of mechanical allodynia by sympathectomy (66). Importantly, TGF-β induces M2-like macrophage polarization (67), indicating that TGF-β-induced M2 macrophage polarization might be responsible for the therapeutic effect of local sympathectomy in the aforementioned study.

Interestingly, cultured M2 macrophages contained and released higher amounts of opioid peptides (63). Similar, a recent study demonstrated that IL-4 application at injured nerves shifted macrophage polarization from a proinflammatory M1 to an anti-inflammatory M2 phenotype. These M2 macrophages continuously synthesized opioid peptides. IL-4 administration further resulted in a long-lasting attenuation of neuropathy-induced mechanical hypersensitivity after discontinuing treatment. Confirming the importance of M2 macrophage-secreted opioids, IL-4-induced analgesia was decreased after neutralizing opioid peptides or blocking opioid receptors at the injured nerves (68). These studies indicate that M2 polarized macrophages may regulate pain perception by modulation of the opioid system. A key observation of our study showing that TNFR2 agonist treatment promotes long-lasting pain recovery was a repolarization of CNS-infiltrating macrophages into an anti-inflammatory M2-like phenotype (18). However, the contribution of M2 polarized macrophages in the spinal cord to pain alleviation and a potential role of the opioid system is not clear yet.

## Immune-Mediated Sex Differences Impact CNP Development

Next to social and psychological factors, functional differences in the immune system contribute to a higher female prevalence for CNP development (69, 70). Using rodent models of injury-induced CNP, it was shown that male and female mice use different immune cells to initiation and maintain CNP. In particular, microglia were shown to be the driver of male neuroinflammation and CNP, whereas T cells primarily drive neuroinflammation and CNP in females (71). These differences seem to be dependent on cell populations, differences in suppression by hormones, and disparate cellular responses in males and females (72). Interestingly, in the absence of adaptive immune cells, e.g., in Rag1^−/−^ mice, female mice use the male, glial-dependent pathway (71). Since sex differences may impact the effectivity of analgesic therapeutics, the different impact of immune cells to pain responses needs to be considered in therapeutics development. Indeed, we observed different responsiveness of male and female mice in a preclinical trial for a novel TNF modulating compound (73). Therefore, it is important to study the therapeutic responses in males and females during preclinical evaluation, in particular if they address T cell or microglial responses. Therapies based on Treg modulation work across sexes (18) indicating that they may interfere with microglial and adaptive immune cell contribution to CNP development and maintenance.

## Conclusion and Outlook

**Figure 1:**
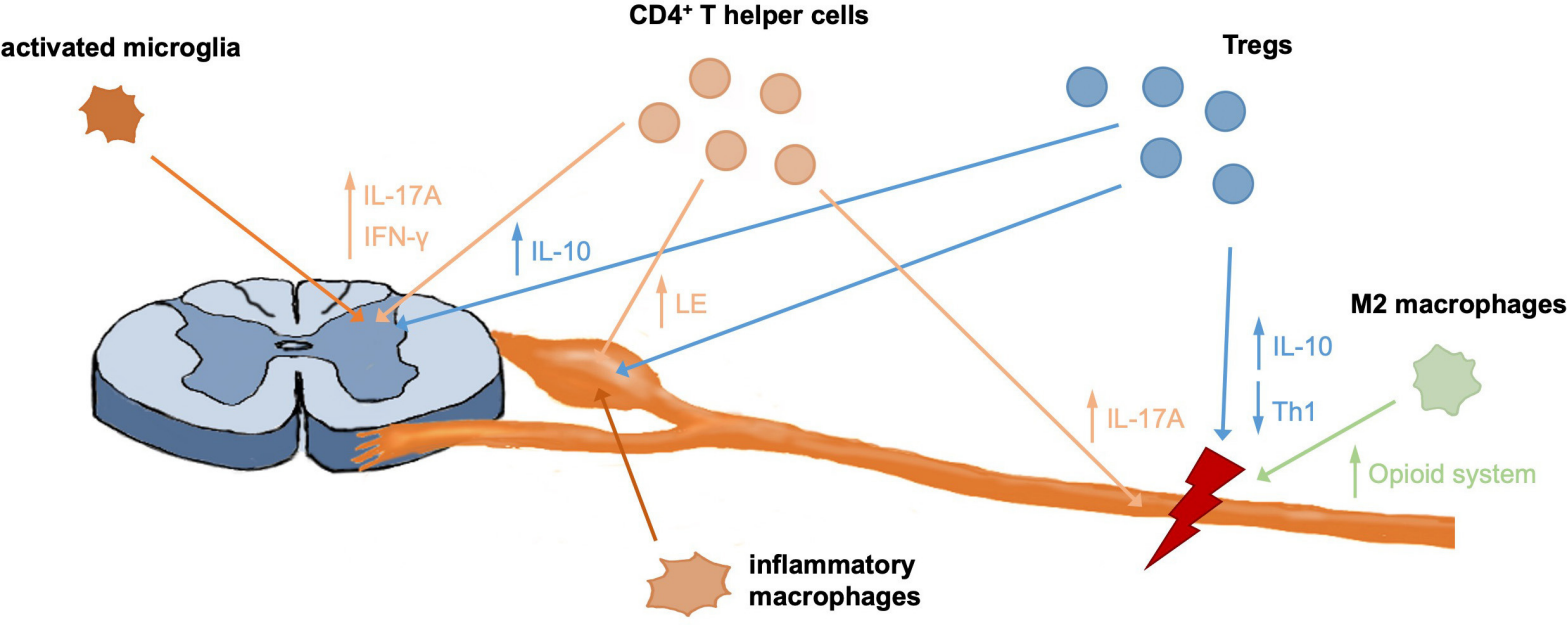
Contribution of different immune cells to pain development and recovery. CD4^+^ T helper cells were shown to infiltrate the spinal cord, DRG and injured nerve, where they contribute to pain responses through different mechanisms. Further, DRG invading macrophages were shown to be important mediators of pain. Like T effector cells, Tregs infiltrate the nerve, DRG, and spinal cord in neuropathic mice and contribute to immunomodulation and tissue regeneration through different mechanisms, including secretion of anti-inflammatory IL-10. M2 macrophages were shown to initiate analgesic responses in the nerve through upregulation of the endogenous opioid system and anti-inflammatory responses.

The contribution of immunity to development and maintenance of CNP are well-established and a complex interaction of different immune cells contributes to CNP development (Figure 1). Over the last years a growing body of literature on the protective and regenerative role of the immune system for pain has been published, including contributions of CD8^+^ T cells, Tregs and M2 macrophages (Figure 1). Modulation of immune responses, e.g., by targeting inflammatory or anti-inflammatory mediators of peripheral immune cells, therefore is a promising therapeutic approach to alleviate neuropathic pain (Table 1). Concluding, a detailed understanding of immune-mediated tissue regeneration in pain may promote the development of novel immunotherapies for pain alleviation and ultimately may translate into novel non-opioid therapies.

**Table 1:** Overview of peripheral immune cell contribution to pain development and recovery.

**Immune cell**	**Role**	**Mediators (Therapeutic target)**
CD4^+^ Th1 cells	Promote pain development and maintenance	IL17A, leukocyte elastase
CD4^+^ Th2 cells	Promote pain recovery	IL-10, IL-4
CD8^+^ T cells (educated)	Promote pain recovery	IL-10
Tregs	Promote pain recovery	IL-10, IL-35, TGF-β, TNFR2
Inflammatory macrophages	Promote pain development and maintenance	CX3CL1, ROS
Anti-inflammatory macrophages	Alleviate pain	Endogenous opioids, TGF-β, IL-10

## Author Contributions

RF wrote the review and generated the figure. JB reviewed and revised the manuscript. Both authors contributed to the article and approved the submitted version.

## Conflict of Interest

JB and RF are named inventors on patent applications covering the use of TNFR2 agonists. RF is a named inventor on patent applications covering the TNFR2 agonist technology.
